# Immunological Role of the Maternal Uterine Microbiome in Pregnancy: Pregnancies Pathologies and Alterated Microbiota

**DOI:** 10.3389/fimmu.2019.02823

**Published:** 2020-01-08

**Authors:** Jonah Bardos, Desiree Fiorentino, Ryan E. Longman, Michael Paidas

**Affiliations:** ^1^Department of Obstetrics and Gynecology, Miller School of Medicine, University of Miami, Miami, FL, United States; ^2^Division of Clinical and Translational Genetics, Department of Human Genetics, Miller School of Medicine, University of Miami, Miami, FL, United States

**Keywords:** uterine microbiome, pregnancy failure, pre-eclampsia, IUGR, early pregnancy immunology, endometrial micorbiome

## Abstract

Understanding what happens at the time of embryo implantation has been the subject of significant research. Investigators from many differing fields including maternal fetal medicine, microbiology, genetics, reproductive endocrinology and immunology have all been studying the moment the embryo interacts with the maternal endometrium. A perfect relationship between the uterus and the embryo, mediated by a tightly controlled interaction between the embryo and the endometrium, is required for successful implantation. Any factors affecting this communication, such as altered microbiome may lead to poor reproductive outcomes. Current theories suggest that altered microbiota may trigger an inflammatory response in the endometrium that affects the success of embryo implantation, as inflammatory mediators are tightly regulated during the adhesion of the blastocyst to the epithelial endometrial wall. In this review, we will highlight the various microbiome found during the periconceptual period, the microbiomes interaction with immunological responses surrounding the time of implantation, its effect on implantation, placentation and ultimately maternal and neonatal outcomes.

## Introduction

The human body is colonized with over ten times more bacteria than the number of cells (1). Most prior medical research has been focused on disease causing bacteria and only recently, has there been a coordinated focus on studying the resident bacteria, viruses, and fungi collectively called the microbiome. In 2008, the NIH undertook a large human microbiome project in an effort to characterize all the microorganisms living in association with the human body in 300 healthy volunteers (2). The focus was on nasal passages, oral cavity, skin, GI tract, and the urogenital tract including the vagina. Few studies have focused on uterine microbiome as it was originally thought to be a sterile environment. Prior microbiome studies utilized either culture or 16S sequence-based technology in determining the bacterial environment. Early work describing the reproductive microbiome came from culture-based approaches (3). However, data from the next generation sequencing of the vaginal microbiome show that many organisms are not identified when culture only based approaches are utilized (4). Early studies suggested only about 1–2% of bacteria that is present can be picked up on culture (5), while recent studies using next generation sequencing suggest 20–60% depending on body site (1). More recent reproductive studies utilized 16S RNA hypervariable region gene sequencing, which can determine the genus and species level of bacteria, but not sub-speciation (6). There are both advantages and disadvantages to 16S sequencing. 16S is significantly cheaper and can be performed on smaller amount of DNA sample since an amplification process is required. However, the amplification process can also introduce an inherent amplification bias making it harder to accurately determine relative abundance of the bacterial species. Additionally, 16S technology utilizes a mapping database, meaning the species must have been characterized before by others. When compared with 16S-based sequencing, shotgun metagenomics can help with the identification of lower taxonomic resolution meaning detecting low abundance microbial communities and can better differentiate between closely related species (7). Essentially, prior work with culture and 16S was potentially missing key factors of the reproductive tract microbiome, however there are no studies to date published on shotgun sequencing of the endometrium at the time of an embryo transfer.

Recently there has been a new focus on determining uterine microbiome after a pilot study in 2016 showed that women with a non-lactobacillus dominant (NLD) uterine environment had an almost 40% drop off in pregnancy rates (8). Another study in 2015, utilized targeted sequencing and microarray data focused on the hypervariable regions of 16S rRNA and showed that uterine microbiome at the time of transfer can be characterized by sequencing the tip of the transfer catheter from the embryo transfer (9). Since then, many authors have been attempting to characterize the natural microbiome of the uterus to determine what affect the uterine microbiome has on pregnancy outcomes (10). Current theories suggest that NLD microbiota may trigger an inflammatory response in the endometrium that affects the success of embryo implantation, as inflammatory mediators are closely regulated during the adhesion of the blastocyst to the epithelial endometrial wall.

A perfect relationship between the uterus and the embryo, mediated by a tightly controlled interaction between the embryo and the endometrium, is required for successful implantation. With almost 4 million births annually it is important to continue researching and discovering key factors of healthy pregnancies. Any factors affecting this communication, such as altered microbiome may lead to poor reproductive outcomes.

## Normal Microbiome

### Uterine Microbiome

For many decades, the uterus was thought to be a sterile environment. Despite being adjacent to the bacterially colonized vagina, it was thought that the cervical mucous maintains uterine sterility. It wasn't until recently that this dogma was challenged. In 1996, Egbase et al. demonstrated that the reproductive tract microbiota can have an effect on IVF outcomes (11).

Is there a healthy “normal” uterine microbiome? This question has been hard to answer. Over the last 15 years about 10 studies have examined uterine microbiome, and over 60 have looked at the reproductive tract microbiome, however most studies to date involved women with pathology (12, 13). Prior to 2014 most studies had attempted to utilize culture techniques which has been shown to miss the majority of the pathogens present, as only bacteria whose metabolic needs are met will grow (14). Additionally, highly abundant and fast growing bacteria will dominate and suppress others (15). Thus, it was not until the expansion of next generation sequencing that a better picture of the reproductive microbiome was even possible. In 2015, Franasiak et al. was the first to measure uterine microbiome from catheter tips at the time of an embryo transfer (6). In the studies of healthy women the most consistent phyla have been Firmicutes, Bacteroidetes proteobacteria, and actinobacteria (6, 8–10, 16–20). The most common genera found in multiple studies have been *Lactobacillus* and *Streptococcus* both of which can be found in the vagina and cervix (12). Some studies have found that *Lactobacillus* is more prominent in women with endometrial polyps or chronic endometritis (21). Multiple studies have suggested that chronic endometritis is associated with recurrent pregnancy loss (22, 23). Chronic endometritis (CE) is typically defined as a chronic inflammation of the uterine lining and is associated with the presence of plasma cells on endometrial biopsy (24, 25). Numerous microbes have been found in patients with CE including *Gonorrhea, Chlamydia, Escherichia coli, Streptococcus, Staphylococcus, Enterococcus fecalis*, and non-microbial causes, such as retained tissue. Additionally, endometriosis has been hypothesized to alter the endometrium through increased inflammation and progesterone resistance which can affect implantation, increase risk of miscarriage, poor pregnancy outcomes including pregnancy induce hypertension and preterm birth (26). It would appear that increased inflammation at the endometrial level regardless of the cause may affect implantation and pregnancy outcomes.

A landmark study by Moreno et al. in 2016 suggested that *Lactobacillus* dominance (>90%) conferred a protective benefit resulting in increasing implantation rates (8). However, given that only 16S methodology was used it is unclear whether certain species or subspecies of *Lactobacillus* may be capable of conferring this benefit. A recent study on the endometrial microbiota and chronic endometritis reported that *Lactobacillus crispatus* was less abundant in patients with CE suggesting that there may be certain *Lactobacillus* spp that is protective (27). More comprehensive whole genome shotgun sequencing (WGS) may help answer this question.

Where does the uterine microbiome come from? There are currently a few theories. The primary theory is ascension from the vagina. While there is a known cervical plug that does protect the uterine environment, we know that, during intercourse, semen is able to ascend into the uterus through small channels in the cervical mucus. Studies have shown evidence of a uterine pump moving radio tagged isotopes from the vagina into the uterus within 15 min of intercourse (28). Other possible methods include hematogenous spread from the gut and transmembrane gut leakage into the peritoneal cavity with retrograde ascension via the fallopian tubes. Dendritic cells and leukocytes traffic bacteria found in the gut and can hematogenously spread bacteria to other locations, such as the uterus (29). One study showed that when genetically labeled *Enterococcus fecium* was placed in the oral cavity of a mouse it could be detected in the placenta (30). Given these possible origins of the uterine microbiome, it is important to understand the microbiome of various anatomical locations.

### Vaginal Microbiome

Since the most likely explanation is ascension, it is important to spend some time understanding the vaginal microbiome. Over the last decade studies involving both 16S and metagenomics have examined the microbiome of the human vagina. The human vagina has been shown to harbor predominantly *Lactobacillus* spp in concentrations as high as 10^7^−10^9^ per gram of vaginal fluid (10, 31, 32). The high levels of *Lactobacillus* are known to secrete lactic acid creating the characteristic low PH found in the vagina. This low PH has been shown to help protect against cervico-vaginal infections (33, 34). While the exact reason for *Lactobacillus* predominance is not known, there are some beneficial aspects of eubiosis. One benefit of the native microbiome is a concept called competitive exclusion. Competitive exclusion is where native microbiome can adapt to be the best nutrient scavenger in that environment, competing with potential invaders for nutrients and in turn starving other pathogens. In reproductive aged women there are five major types of vaginal microbiota or community state types (CST). CST I II, III, and V are all predominantly *Lactobacillus*. Whereas, CST IV is predominantly a mixture of strict and facultative anaerobes including *Gardnerella, Atopobium, Mobiluncus*, and *Prevotella* (35, 36). CST IV is commonly broken down into CST IV-A and CST IV-B which is associated with bacterial vaginitis (BV) (36). CST I appears to be more common in Caucasian women and protective against BV, while CST IV is more common in African American and Hispanic women (37, 38).

Studies have suggested that the vaginal microbiota is subject to frequent fluctuations (39). In some women, menses or sexual behaviors may trigger transitions between the CSTs at different points in time (36). During times of elevated estrogen, such as immediately prior to ovulation, *Lactobacillus* tends to stabilize, while during menstruation *Lactobacillus* tends to decrease (40). While menses appears to alter the composition of the vaginal microbiome, the change appears to depend on the initial CST and other factors, such as the use of pads or tampons (41). The above studies suggest the dynamic nature of the vaginal microbiome, questioning whether one can reliability predict microbiome between menstrual cycles.

There is debate in the literature regarding whether contraceptive options have an effect on the vaginal microbiome. One study suggested that hormonal contraceptives did not have an effect on vaginal microbiota while copper IUDs are associated with an increase in BV (42). Another study suggested that women who took combined oral contraceptives were more likely to have *Lactobacillus* dominance compared to women who used barrier methods (43). One study suggested that LNG-IUD may increase the risk of candida and decrease *Lactobacillus* dominance (44). Still another study suggested it was not contraceptive options which drove vaginal microbiome changes but rather the number of sexual partners and the woman's ethnicity (37). Additionally, elevated hormones at the time of implantation could affect the microbiome, for example during a fresh embryo transfer.

A number of other conditions have been associated with an altered vaginal microbiome. Some studies have suggested that *Lactobacillus* dominated microbiota can be protective against PID (32). CST I appears to decrease the risk of STIs, such has *Chlamydia*, while CST III appears to be associated with an increased risk of *Chlamydia* infection (45). CSTs II and V appear to compete with Gonorrhea thereby possibly decreasing the risk of infection (46).

Alterations of the vaginal microbiota may also be responsible for various pregnancy outcomes. During pregnancy menses cease and there is a consistently elevated level of estrogen with a concomitant increase in *Lactobacillus* dominance (47). Several studies have suggested that altered microbiota are associated with preterm birth (48–50). Preterm premature rupture of membranes (PPROM), chorioamnionitis and early or late miscarriages have also been associated with changes in the vaginal microbiome (51–56). One study of the vaginal microbiome on the day of embryo transfer suggested that a lower microbiota index is associated with better IVF outcomes. A recent study suggested that higher levels of vaginal *L. crispatus* is associated with higher chance of pregnancy when utilizing ICSI (57). Neither of these studies included sampling of the upper reproductive tract; therefore, it is hard to draw conclusive results (58). The literature appears to support the concept that alterations in the vaginal microbiome are associated with various poor outcomes and that examining the vaginal along with the upper reproductive tract microbiome at the time of an IVF transfer may shed additional information into the causes of these poor outcomes.

### Placental Microbiome?

Numerous studies have suggested that bacterial infections are the cause of PPROM and preterm labor (59–61). However, until 2014, it was thought that the placenta did not contain its own microbiome. In chorioamnionitis, an inflammatory infectious process of the fetal side of placenta, the most commonly isolated pathogens are *Bacteroides* species, *E. coli, Gardnerella vaginalis, Mycoplasma hominis, Peptostreptococci, Streptococci*, and *Ureaplasma urealyticum* (62). This would suggest that pathologic bacteria can invade the amnion, and chorion from the vagina. Additionally, there are theories of oral placental transmission in patients with poor periodontal disease associated with poor pregnancy outcomes (63). However, the idea that a placenta contains its own healthy microbiome only came about recently after the seminal publication by Aagaard et al. in 2014 (64). This study found multiple phyla in the placental microbiome namely *Firmicutes, Tenericutes, Proteobacteria, Bacteroidetes*, and *Fusobacteria*. The initial results were called into question as the techniques did not account for live vs. dead bacteria and did not provide a maternal blood sample to determine if the microbiome was from the maternal villi or from the fetal side (65). Additional studies utilizing high throughput sequencing technology confirmed the presence of a placental microbiome (66, 67). Given that the prevailing opinion for many years was that the uterus was sterile, there were theories proposed that the findings were contaminations in either technique or processing. Later studies suggested that DNA reagent kits have their own distinct microbiome called a “kitome” (68). Researchers suggested that, in locations with a high biomass, such as the gut, low level contamination of the “kitome” would not be detected, which would explain why many of the gut studies did not have this issue. However, when looking at ultra-low biomass locations, such as the placenta or uterus, the results may represent the kit without proper controls (69). A recent seminal study in which the researchers carefully controlled for possible contaminants by utilizing multiple detection methods including culture, qPCR, 16S rRNA gene sequencing and shotgun metagenomics, demonstrated that no resident microbiota could be identified in the placenta. While this study was done with the best technology currently available, if a uterine microbiome exists, as many studies have suggested, it is highly unlikely that the placental and uterine microbiome are not related. It is possible that the placental microbiome is currently undetectable due to ultra-low biomass or the limitations of current technology, however dismissing it as non-existent may be premature.

## Normal and Abnormal Immunological Responses to Pregnancy

The embryo is initially fertilized in the fallopian tube and must evade the immune system, grow and move into the uterus before interacting with the endometrium for implantation. The process of implantation is divided into three key steps: apposition, attachment (adhesion), and penetration (invasion). The immune system plays a role in each step of the embryo's development through the delivery of a live healthy fetus.

An egg is released as a maternal cell containing maternal antigens and would be recognized by the immune system as “self.” Sperm then enters the egg typically in the ampullary region of the fallopian tube, triggering multiple changes including production of its own antigens comprised of both maternal and paternal components. Until the embryo starts presenting its own foreign antigens it is viewed as a maternal cell. Once it starts presenting its own antigens it is surrounded by the zona pellucida, a hard shell protecting the embryo from maternal immune cells. Additionally, there are maternal cumulus oophorus cells that provide some protection for the first few days. After the first few days, the embryo must interact with the maternal system. Given that donor embryos are able to implant in surrogates, there must be communication from the embryo that ultimately prevents the maternal immune system from attacking it. It would appear that this communication does not occur immediately given that it takes 4–5 days post-embryo transfer for implantation to occur. During that time a series of events occur, including endometrial priming, immune tolerance and ultimately implantation.

The immune system is usually thought of as a mechanism for the body to defend itself against invaders. Although the egg comes from the woman and would be considered “self,” the sperm displays paternal antigens. Shortly after conception the fetus begins displaying major histocompatibility complex (MHC) genes that are a combination of both mother (self) and father (foreign) (70). How then are embryos not attacked and destroyed by the immune system in every pregnancy? The immune system plays an important role in pregnancy success. A combination of multiple strategies are employed to selectively circumvent or engage different aspects of the immune response.

The endometrium has an innate immune system called pattern recognition receptors (PRR) that can detect certain pathogenic receptor patterns locally and mount an inflammatory response. Examples include toll like receptors 1–10, Nod like receptors, and others (71). Some have suggested that local bacteria can also use the PRR as a way to communicate with the host and induce a safe environment (72). A prime example is the way that T-cells are differentiated.

### T-Cells

There are many different types of T cells that are part of the adaptive immune response. T-helper cells (Th) start out from the same precursor as a Th0 cell. Dendritic cells, a type of antigen producing cell (APC), presents antigens to the naïve Th0 cells and depending on the environmental milieu of cytokines, chemokines, and bacteria the Th0 will differentiate into one of 4 main cell types: Th1, Th2, Th17, and T-regulatory (Treg) (73). Th1 cells are stimulated due to the presence of bacterial DNA and produce pro-inflammatory cytokines, such as TNF and IL12. Th2 cells are stimulated by IL4, IL6, IL10, and IL11 and produce anti-inflammatory cytokines, such as IL4 (74). Th17 is induced based on TGFβ, IL6, and IL21 and produces pro inflammatory cytokines (74). Treg cells are induced by TCR and TGFβ, express Foxp3, CD25 and are key players in inducing tolerance and negative regulation of immune-mediated inflammation (75). Local microbiome can affect whether the TGFβ increased Th17 or Treg cell population (76). Treg cell dysfunction is associated with autoimmune, auto-inflammatory disorders, allergies, as well as acute and chronic infections (77).

The embryo must convince the body that it should be tolerated. Treg cells take “non-self” antigens that are presented to it and promote active tolerance to those antigens by down-regulating the Th1 and Th17 responses (70, 78, 79). Treg cells inhibit additional T-cell proliferation and proinflammatory cytokine productions by producing TGFβ and IL10 while suppressing B cell proliferation, antibody production, NK cell cytotoxicity, as well as dendritic and macrophage maturation and activation (80–82).

Treg cells help down-regulate immune response against the embryo to allow for intimate interaction with the endometrial lining (70). Treg cells are recruited to the uterus shortly after conception which is an important time for the fledgling embryo's existence. Apposition, implantation and early placental morphogenesis is a key time in which the embryo begins to interact directly with the maternal immune system. Poor immune tolerance at this point could result in shallow implantation which has been implicated as a possible cause of IUGR and miscarriage (83, 84). Additionally, problems in early placental development have been linked with pre-eclampsia, an increasingly common morbidity of late pregnancy (85). There is evidence that the local microbiome can induce a shift to Th1 and away from Treg cell dominance. For example, *Bacteroides* is thought to increase the presence of Th1 cells (86). An upregulation of Th1 will cause an increase in local inflammation and an augmented local immune response. As mentioned prior, it would appear that increased inflammation at the endometrial level regardless of the cause may affect implantation and pregnancy outcomes. However, beneficial microbiome can increase uterine Treg cells locally thereby downregulating the immune response and inducing tolerance to a specific species or a pregnancy (13).

### Blast Development

*In vivo*, in the first 48 h post-fertilization the embryo is encased in a hard-outer shell called the zona pellucida. Within 2 days, the embryo moves from the fallopian tubes into the uterus where it floats while undergoing cleavage and differentiation into a blastocyst. About 5–6 days post-fertilization, the embryo hatches from the zona pellucida exposing its outer trophoblasts to the endometrial lining (87). Embryos display cytokine receptors from conception until implantation. Cytokines play a key role in embryo development, gene expression, cell number, embryo competence and viability (88, 89). In healthy women, embryotrophic factors, such as GM-CSF, CSF1, LIF, HB-EGF, IGF1, and IGF2 all act to promote blastocyst development, increase blast cell numbers and gene expression. Decreased cell number and poor trophoblast development can cause changes in placental structure and nutrient transport functions leading to pregnancy loss or developmental damage (90, 91). GM-CSF is produced by the uterine epithelial cells, exerting a pro survival and anti-stress effect required for fetal viability and offspring health (92). Embryotoxic cytokines, such as TNF, IFNG, and TRAIL are stimulated during inflammation or local infections via toll like receptors and induce apoptosis and inhibit embryo development (93). Other environmental factors, such as hyperglycemia and obesity can also induce embryotoxic chemokines which may explain why these conditions are associated with poor pregnancy outcomes. This complex balancing act between embryotrophic vs. embryotoxic cytokines acts a physiologic control enabling pregnancy when the uterus is under optimal conditions and inhibiting pregnancy when the conditions are sub-optimal. Obviously, these alone are not the only determinants, as obese diabetic women do get pregnant, however, the delicate cytokine balance, controlled by many factors including the local microbiome, can have a large impact on reproductive outcomes.

### Endometrial Receptivity

Sex steroids also play an important role in modifying the immune response and preparing the uterine lining for implantation. In the proliferative phase, estrogen stimulates proliferation and differentiation of endometrial cells as well as pro inflammatory cytokines, such as CSF1, GM-CSF, INFG, and TNF. During the luteal phase, progesterone stimulates the mesenchymal cells to prepare for implantation and suppresses the pro inflammatory cytokines by inhibiting epithelial cell productions of GM-CSF 1 and IL1. Progesterone also enhances the expression of IL8 and attracts uterine NK cells (70). Estrogen increases Treg attracting chemokines, CCL3, 4, and 5, while progesterone sustains the Treg population and increases their suppressive function (94). Additional endometrial chemokines, such as LIF and IL11 are essential for decidual and vascular changes required to allow proper trophoblastic invasion (95). It has been suggested that in patients with endometriosis the endometrial tissue may have progesterone resistance which could explain the increased inflammation and poor pregnancy outcomes (96). Placental trophoblasts secrete TGFβ which helps suppress immunity, and assists in inducing Treg cells at the placental maternal interface (70, 97). Excess inflammatory cytokines, such as TNF, IFNG, and IL2, during the implantation period can act to skew the adaptive immune response away from Treg cells and toward cytotoxicity (98). Excess inflammatory factors are found during local infection and times of nutritional or metabolic stress, thereby helping the body suppress pregnancy during unfavorable conditions; leading to failed implantation or pregnancy loss. Excess inflammation may also be found when the host microbiome is altered thereby conferring negative effects on implantation and outcomes.

Other cells also play a role in controlling immune response at the uterine level. Uterine macrophages produce Treg stimulatory cytokines TGFβ, PGE2, and IL10 (99). Decidual macrophages also secrete matrix proteinases which can help with trophoblast invasion (100). Dendritic cells present antigens to naive T cells to stimulate Treg cell activation before implantation (101). Uterine NK cells are involved in the endometrial remodeling needed for implantation (102). Seminal fluid appears to play a role in driving leukocytes to the uterus. Seminal fluid can stimulate the expansion of the Treg population via TGFβ and PGE2 along with paternal antigens and help inhibit the activated Treg cells from leaving the uterus, inducing tolerance to the embryo.

The embryo also plays a role in its own fate by secreting factors. PreImplantation Factor (PIF) is secreted only by viable embryos and can be measured in embryo culture media. PIF can be seen in maternal circulation as soon as 4 days post-embryo transfer indicating that it does come from the embryo. When PIF is seen in maternal plasma, the chance of normal pregnancy is significantly higher (103). PIF appears to have anti inflammatory effects. It provokes global immune regulation by binding ligands to CD14 monocytes/neutrophils and to T and B cells promoting the required Th2/Th1 cytokine ratio. It also appears to affect genes involved in oxidative stress, protein misfolding, and platelet activation.

Human chorionic gonadotropin (hCG) has many different roles during early pregnancy including immunosuppression. Its' most well-known and crucial role is to promote corpus luteal progesterone production for the first 3–4 weeks after implantation (104). In addition, hCG is thought to help promote placentation and angiogenesis by recruiting and promoting VegF and Treg function necessary for decreasing local inflammation (105).

The immune environment during the peri-conceptual period is controlled by many factors, including cytokines, chemokines and leukocyte lineages. These factors are intertwined with ovarian steroid hormones, seminal fluid, diet, nutrition, metabolism, obesity, infections, and the microbiome to influence the balance between embryotrophic and embryotoxic milieus and ultimately a successful or unsuccessful pregnancy.

## Uterine Embryo Signaling and Implantation

As mentioned previously there are three stages to implantation: apposition, adhesion, and invasion. We have discussed how the microbiome cytokine influence can affect the competency of the embryo. We will now briefly discuss each stage of implantation to understand how the microbiome could help or hinder the successful progression of pregnancy. Researchers still do not fully understand how implantation works. Decidualization is the transformation of the endometrium into a receptive state prior to the presence of the embryo. Between 6 and 10 days after ovulation the point in which the endometrium is receptive occurs, called the window of implantation. Once the embryo reaches the primed endometrium there is a process of selection, apposition, attachment, implantation, and ultimately placentation that requires intricate embryo uterine interactions to occur in a synchronized fashion for pregnancy to be sustained. While the main drivers of these processes are hormonal control, additional local cytokine factors may play an important role. Alterations from the natural microbiome may affect the local cytokine/chemokine profile thereby affecting implantation.

### Apposition and Adhesion

Approximately 6–7 days after fertilization the blastocyst makes contact with the uterine wall in a transient dynamic process called apposition. Microvilli extending from the syncytiotrophoblast interact with small microprotrusions from the uterine epithelial cells called pinopodes. This step is unstable and must occur first before the embryo can firmly attach to the wall. The interaction between the uterine cell wall and the embryo activates cytokine signaling and remodeling of the cytoskeleton of the epithelial layer. Decidual macrophages secrete matrix proteinases which can help with trophoblast invasion (100). Matrix metalloproteinases are important players in trophoblast invasion and are regulated by cytokines and tissue inhibitors (TIMPs) (106). TIMPs are upregulated by TGFβ inhibiting proper matrix degradation which can affect implantation (107). Increased TGFβ can be caused by colonizing microbes (76). Tight junctions are disrupted mediated by local cellular communication (108). Uterine NK cells are also involved in the endometrial remodeling needed for implantation (109).

### Invasion

The final stage is invasion during which the syncytiotrophoblast invade the uterine epithelium. By approximately day 10 after fertilization, the blastocyst is surrounded by uterine tissue and the epithelium regrows over the implantation site. The cytotrophoblasts continue to expand until they reach the myometrium and the uterine spiral arteries leading to the establishment of uteroplacental circulation (110). The resulting uterine arterioles are composed of both maternal and fetal cells, underscoring the importance of immune tolerance in proper placentation. Implantation is the result of an intricate bi-directional dialogue between the embryo and endometrium mediated by a host of factors regulating the invading cells from cytokines and growth factors to steroid hormones and proteinases (111). Dysfunctional placentation can have clinical implications due to either excessive or inadequate invasion. Excessive invasion of the cytotrophoblasts can lead to abnormally strong attachment or a morbidly adherent placenta, such as an accrete, increta, or percreta depending on depth of invasion. Inadequate invasion has been implicated in pre-eclampsia, and IUGR (112, 113).

## Current Understanding of Pre-Eclampsia

After years of study, the mechanisms by which pregnancy incites or aggravates hypertension remain unknown and hypertensive disorders continue to play an important role in maternal morbidity and mortality worldwide. Preeclampsia is more likely to occur in women who are exposed to chorionic villi for the first time (nulliparous women); are genetically predisposed to hypertensive disorders of pregnancy; have preexisting conditions associated with endothelial cell activation or inflammation including diabetes, cardiovascular or renal disease, or immunologic disorders; and in women who are exposed to a superabundance of chorionic villi (as in the cases of twins or molar pregnancies). Additionally, while a fetus is not required for the development of preeclampsia (as in molar pregnancies), the presence of chorionic villi is. A case series published in 2008, reported that PEC may develop even when the chorionic villi are extra-uterine as in the case of an abdominal pregnancy (114). Regardless of the underlying cause, the events leading to PEC all result in systemic vascular endothelial damage leading to transudation of plasma, vasospasm, and thrombotic sequelae.

Currently, the four most likely explanations for the development of PEC include: immunological maladaptive tolerance between maternal, paternal, and fetal tissues; placental implantation with abnormal trophoblastic invasion; oxidative stressors resulting in endothelial cell dysfunction; or genetic factors including predisposing genes and epigenetic influences.

Loss of maternal immune tolerance to paternally derived antigens is a possible etiology of preeclampsia (115). This hypothesis is supported by the fact that preeclampsia is more likely to occur when the formation of blocking antibodies to paternal antigens might be impaired. For instance, there is an increased risk in first pregnancies or pregnancies with a new partner and molar pregnancies which have an increased paternal antigenic load. Additionally, pregnancies with a fetus with trisomy 13 who have elevated antiangiogenic factors arising from the presence of an extra copy of *soluble fms-like tyrosine kinase 1* which is located on chromosome 13 have increased risk of preeclampsia (116, 117). A recently published study indicated that the angiogenic factors placental growth factor, *soluble fms-like tyrosine kinase 1*, and soluble endoglin are biomarkers with predictive potential for preeclampsia. The soluble *fms-like tyrosine kinase 1*/placental growth factor ratio is able to accurately predict the short term absence of preeclampsia and suggest the likelihood of adverse events within 4 weeks (118). Further, another study from April 2019 demonstrated that CD3^+^, CD8^−^, FoxP3^−^ T cells were associated with uteroplacental acute atherosis which is a common lesion of the maternal spiral arteries in the decidua basalis in preeclampsia (119). The decidua basalis layer forms the maternal-fetal immunologic interface where fetal extra-villous trophoblasts interact with maternal immune cells. Immune maladaptation may also play a role in the pathophysiology of preeclampsia. Extra-villous trophoblasts express HLA-C which is a ligand for killer immunoglobulin-like receptors (KIR) on NK- and T-cells. The combination of maternal KIR-B haplotype and fetal HLA-C2 has been shown to be significantly associated with acute atherosis. Thus, it seems that interactions between fetal HLA and activating KIRs on maternal decidual NK-or T-cells may promote local decidual vascular inflammation and trigger the formation of acute atherosis (120). This supports the theory that inadequate maternal tolerance of invasive trophoblast, which can be due to a shift in the immune system against tolerance, i.e., the Th1/Th2 ratio, can trigger poor trophoblast invasion and the occurrence of preeclampsia (121).

As previously discussed, normal implantation requires invasion of the syncytiotrophoblast into the uterine epithelium, ultimately resulting in remodeling of the uterine spiral arteries to create a dilated low resistance vessel. In pregnancies complicated by preeclampsia, there is thought to be incomplete invasion of the spiral arteriolar wall leading to small caliber, high resistance vessels with correlation shown between the degree of syncytiotrophoblast dysfunction and the severity of the resulting preeclampsia (122, 123). These high resistance vessels impair placental blood flow resulting in diminished perfusion and a hypoxic environment. There is subsequently a release of placenta microparticles which incites a systemic inflammatory response (124, 125).

Decreased placental perfusion from dysfunctional placenta implantation results in repeated ischemia/reperfusion episodes which creates a favorable environment for developing oxidative stress and stimulates the production and secretion of pro-inflammatory cytokines as well as vasoactive compounds. Cytokines, such as tumor necrosis factor alpha and interleukins contribute to systemic oxidative stress which is characterized by reactive oxygen species and free radicals that lead to the formation of lipid peroxides (126). These lipid peroxides then generate highly toxic radicals that injure vascular endothelial cells, modify nitric oxide production by the cells, and interfere with prostaglandin balance. The above cascade results in systemic endothelial dysfunction characterized by vascular inflammation and constriction. Other consequences of oxidative stress include production of lipid-laden macrophage foam cells that are seen in acute atherosis, activation of microvascular coagulation, and increased capillary permeability. The important role of oxidative stress in the pathophysiology of preeclampsia is further supported by a study in which concentrations of maternal oxygen free radical were measured in 52 women with and without preeclampsia. Maternal serum concentrations of oxygen free radicals were significantly increased in the preeclampsia group relative to the normal group (127). Some researchers conclude that oxidative stress appears to be the central component of both placental and endothelial dysfunction, the causative etiology of preeclampsia (128).

Preeclampsia is a multifactorial condition with a strong genetic component. Immune maladaptation, endothelial function, and oxidative stress all encompass genetic factors that could be responsible for the pathogenic changes that take place in preeclampsia. Additionally, there is evidence that paternal genes significantly increase the risk of preeclampsia (129). In a study of ~1.2 million births in Sweden, a genetic association for preeclampsia was noted (130). The hereditary risk of preeclampsia most often cited is 20–40% for daughters of pre-eclamptic mothers and 11–37% for sisters of pre-eclamptic women (131). The hereditary predisposition for preeclampsia most likely results from the interactions of hundreds of genes and it is doubtful that any one gene will be found responsible. Epigenetic alterations have also been noted in preeclampsia including alterations of methylation in the placenta of pre-eclamptic patients. Additionally, it is hypothesized that antiangiogenic and cytotoxic factors released by the placenta in preeclampsia have the potential to induce epigenetic modifications in maternal tissues (132).

## Current Understanding in Causes of IUGR

Fetal growth restriction, also known as intrauterine growth restriction (IUGR), is a common pregnancy complication that has been linked with a variety of adverse perinatal outcomes. The precise mechanism by which normal growth occurs is unknown, but IUGR is usually the end result of maternal, fetal and placental causes or a combination thereof. Although the primary pathophysiologic mechanisms underlying these conditions are different, they often result in the same final common pathway of suboptimal uterine–placental perfusion and fetal nutrition.

Multiple placental, cord, and uterine anomalies are associated with poor fetal growth. Placental insufficiency may be due to abnormal placental development or placental damage. Several placental abnormalities including chronic abruption, infarction, circumvallate shape, chorioangioma, velamentous cord insertion, and umbilical artery thrombosis have been shown to lead to uteroplacental insufficiency and IUGR (133). Inflammation may contribute to placental damage and abnormal development as inflammatory mediators promote thrombosis (133). Hypoperfusion of the placental site may also arise secondary to implantation site disorders. Brosens et al. postulated that there is a partial progesterone resistance in the fetal uterus at the time of birth that may persist into the adolescent years resulting in compromised physiological transformation of the spiral arteries. This theory is supported by the fact that major obstetric syndromes due to impaired placental bed spiral artery remodeling, including preeclampsia, growth restriction, and preterm labor, are all more prevalent in teenage pregnancies (134).

Maternal medical comorbidities, especially those with vascular disease or thrombosis, are also associated with IUGR via poor placental perfusion. Chronic vascular disease, including maternal ischemic heart disease, is associated with higher rates of preeclampsia and IUGR (135). Chronic renal insufficiency is frequently accompanied by underlying hypertension and vascular disease and thus, often also results in IUGR. Additionally, pre-gestational diabetes, especially when complicated by vascular or renal disease, and disease states resulting in chronic uteroplacental hypoxia like pre-eclampsia, chronic hypertension and asthma can lead to significantly reduced birthweight. Maternal conditions, like antiphospholipid syndrome, increase the risk of ischemic placental dysfunction resulting in a similar outcome of poor perfusion and decreased growth (136). Other maternal factors may contribute to IUGR without directly impacting the placenta. These include poor maternal nutrition and eating disorders; constitutionally small mothers, particularly when combined with poor gestational weight gain, and social issues (137–139).

Exposure to drugs and teratogens during pregnancy are associated with IUGR. Cigarette, opiate, alcohol, and cocaine use cause fetal growth restriction directly and by decreasing maternal food intake (140, 141). Even prescription medications like anticonvulsants, antineoplastic agents, and antithrombotic drugs are teratogens and can result in fetal growth restriction (142, 143). Multifetal gestation and fetal malformations are also seen in association with intrauterine growth restriction. In one study of pregnancies complicated by gastroschisis, one-third of neonates had birthweights less than the 10th percentile (143).

Still other etiologies of fetal growth restriction interact with the placental, maternal, and fetal compartments. The most important of these include infections and genetic anomalies. In their text book Maternal Fetal Medicine: Principles and Practice, Creasy et al. postulated that fetal infections account for 5–10% of IUGR with malaria accounting for most cases of infection-related fetal growth restriction worldwide (144, 145). Additionally, rubella, CMV, toxoplasmosis, varicella, and syphilis have all been shown to have a causal relationship with fetal growth restriction (146–149). Under normal conditions, maternal genes have the main influence on birthweight (150). Fetal aneuploidy is responsible for up to 5% of IUGR diagnosed at any point of pregnancy and up to 20% of IUGR diagnosed in the first half of pregnancy (151). Trisomy 13 and 18 are usually associated with more severe IUGR while in Trisomy 21 the growth restriction is typically mild (152). Even confined placental mosaicism is associated with low birth weight and adverse pregnancy outcomes (153). In summary there are many possible etiologies including inflammation and abnormal initial placentation all causing the same final pathway of IUGR.

## How Microbiome Affects Immunity in Other Areas of the Body and Implications for the Uterus

Studies looking at germ free mice revealed that without bacteria there are profound effects on mouse lymphoid tissue, suggesting that bacteria is important for immune development (154). The GI tract is the most studied area of human microbiome to date as the majority of immune system interactions occur within the gut (76). It contains the most abundant population of microorganisms with over 5,000 bacteria taxa (155). The gut microbiome is colonized at birth with initial differences noted depending on mode of delivery (156). Unlike the uterine microbiome that undergoes changes with menses, once a child is about 2.5 years old their microbiome remains relatively stable until age 65 (156). Although it is stable, it can be quickly altered by host factors, such as antibiotics, travel or high fat diets (157). Dysbiosis in the gut has been linked to many diseases, ranging from liver disease and GI cancer to metabolic disease, respiratory disease, mental health disorders and autoimmune disorders (158). The suggested reasoning is that the changes in the microbiome can increase immune system sensitivity to pathogens thereby causing increased inflammation (159). The most potent causes of dysbiosis are pathogens in the gut. There is evidence that altered oral flora, such as during periodontal disease can cause many systemic disorders, such as atherosclerosis and poor pregnancy outcomes (160, 161). Chronic low-level periodontal disease may induce low level chronic systemic inflammation (162). There are theories as to why dysbiosis causes these changes. Alterations in gut microbiota has profound effects on the T-cell mediated autoimmune and inflammatory responses. The gut microbiota is known to provide signaling for proper development, differentiation and epigenetic influences of immune cells (154). Dysbiosis can transition the chemokine profile to cause a Th17 pro inflammatory dominance, which can trigger not only local, but also systemic inflammatory responses and has been linked to Alzheimer's disease (159, 163).

In 2012, Hooper and Macpherson described immunological benefits of host intestinal microbial homeostasis: (1) Host microbiome restricts direct contact between epithelia and pathogenic microbes, (2) Host microbiome anatomically limits the exposure of pathogenic bacteria to the systemic immune system, and (3) Host microbiome can aid in the rapid detection and killing of bacteria upon barrier breach (154).

Some of the suggested theories is that is that host bacteria (microbiome) have the chance to adapt to their environment. The adaptation process allows them to specialize in nutrient utilization thereby depleting nutrients that would otherwise be available for invading pathogens. This process, called colonization resistance, could support the potential importance of a healthy uterine microbiome (164). Additionally, symbiotic bacteria may also compete for the cell's receptors. For example, one study utilizing an *in vitro* model found that the presence of lactobacillus in the reproductive tract prevents gonorrhea from attaching to endometrial cells (46).

The host microbiome may also help support a healthy and intact epithelial barrier thereby preventing pathogen access to the cell. In the gut, studies have suggested that the microbiome can affect epithelial cell differentiation, maintenance and adaptation and modulate epithelial cell permeability (165, 166). Given that the uterus frequently undergoes shedding and regrowth, microbial support could play an important role in maintenance of the epithelial tissue. Conversely, dysbiosis could be an underlying cause of poor endometrial thickening and abnormal placentation.

## Hypothesis for How Altered Microbiome Affects PEC and IUGR

As discussed above, studies have shown that changes in the microbiome can prompt inflammation. For example, specific oral pathogenic bacteria including *Fusobacterium nucleatum, Porphyromonas gingivalis, Filifactor alocis, Campylobacter rectus* are associated with both periodontitis and the development of pregnancy disease (167). The involvement of systemic inflammatory responses in pregnancies complicated by PEC and IUGR has led to the theory that maternal infections may be important factors in the pathogenesis of pregnancy complications. At the base of all possible etiologies of preeclampsia, there exists the same common result of systemic vascular endothelial damage. The remodeling of the spiral arteries in the decidua basalis is a critical step in the establishment of a healthy pregnancy. The decidua basalis is the maternal fetal immunologic interface and it has been shown that local inflammation in area can lead to acute atherosis and poor trophoblast invasion. Additionally, studies have shown that women with asymptomatic bacteriuria, urinary tract infection, and chronic pyelonephritis are at increased risk for preeclampsia (168, 169). Another study by den Hollander et al. found that *Helicobacter pylori*, as a cause of chronic inflammatory conditions, is associated with an increased risk of PEC (170). Further, Li Juan et al. demonstrated that preeclampsia is associated with a disrupted gut microbiota composition compared with that of women who had uncomplicated pregnancies (171). In a recent review of the current knowledge about the possible association between the microbiome and the development of preeclampsia, Dunn et al. did a comprehensive literature search and reported that overall, five groups of investigators studied the microbiome of PEC (172). In two of the studies, the placenta site was analyzed; in the remaining three, the mouth, gut, or an intra-amniotic site was examined. Some findings supported the association between pathogenic bacteria and PEC, but specific pathogenic organisms were not identified and further research is warranted. In a 2015 study, placental tissue samples from women with and without preeclampsia were collected and screened for the presence of bacteria by PCR for 16s rRNA and next generation sequencing. 12.7% of the tissue from women with PEC was PCR positive, while all of the placentas of the control group were negative (173).

While the etiology of growth restriction has also not been elucidated precisely, it seems to result from poor uterine-placental perfusion. It is known that inflammation may contribute to placental damage and that any maternal disease state that can lead to utero-placental hypoxia, like preeclampsia, can result in the development of intrauterine growth restriction. In this way, it is possible that the maternal microbiome could modulate the development of growth restriction by influencing the inflammatory state of the placenta and uterus. One example of systemic inflammation contributing to the development of pregnancy complications can be seen in the study of Den Hollander et al. They reported that *H. pylori* seropositivity with CagA-positive strains, which are associated with higher levels of systemic inflammation than CagA-negative strains, is associated with IUGR (170). Additionally, a study analyzing the characteristics of gut microbiota in IUGR and normal birth weight piglets in the first 12 h of life found an imbalanced inflammatory and plasma metabolome profile in the IUGR piglets (174). The gut microbiome is believed to be colonized at birth with differences seen based on mode of delivery and thus, presumably, exposure to the maternal microbiome. To our knowledge no studies have been done, to date, evaluating the uterine microbiome at the time of implantation and its effect on the development of obstetrical complications of pregnancy including preeclampsia and growth restriction.

## Summary of Effects of Microbiome on Uterine Immune System

Based on both *in vitro* mouse and gut studies there are a likely four possible ways in which local microbiome can affect the clinical sequalae of pregnancy. It is possible that the microbiome cause alterations in regional signaling pathways. For example, as mentioned before, alterated microbiome can cause an abnormal inflammatory response through the uterine toll like receptors. When these receptors are activated they can alter the cytokine milieu swaying the local response to a pro inflammatory and anti-tolerance immune cell response. A second method as previously mentioned can be through the alteration of the endometrial epithelial barrier integrity. Certain pathogens cause a localized decrease in matrix degradation proteins which may affect placentation. A third possible method is that the local microbiome has a competitive advantage, as it has adapted to be the best nutrient scavenger in that area and can usually starve out possible invading species in a process called competitive exclusion. Lastly, microbes can secrete metabolites, such as short chain fatty acids that suppress growth of certain species. Taken together, the natural microbiome can have an impressive effect on the local interactions between the embryo and endometrium with implications on implantation, placentation and embryonic growth, ultimately affecting pregnancy and pregnancy outcomes.

## Conclusion–Future Areas of Research

Current data has yet to support what the characterization of a normal uterine microbiome. However, it is hard to believe that a mucosa that is in close proximity to the vagina with a well-characterized microbiome with regular sperm penetration that microbes do not get through the cervix. The question remains as to whether those microbes are transient and whether a host microbiome controls that transience. Prior studies have been limited so far by 16S only sequencing with an amplification bias and potentially missing any species not currently in the bioinformatics database. It is highly likely that a normal endometrial microbiome does exist. It is possible that it is of very low level and therefore easily subject to sampling contamination which could explain why we have not yet discovered the “normal microbiome,” however it is likely present and given what we know about gut microbiome, it likely plays a role in immunoregulation, endometrial remodeling, pregnancy implantation and placentation. Future research should include sampling of multiple possible contamination sites as well as utilizing shotgun metagenomics for a better understanding of all the pathogens at play.

## Author Contributions

JB and DF were responsible for the manuscript preparation. RL and MP were responsible for the important editorial input.

### Conflict of Interest

The authors declare that the research was conducted in the absence of any commercial or financial relationships that could be construed as a potential conflict of interest.
